# The Flow of Healthcare Information in Rural and Remote Settings: A Qualitative Approach

**DOI:** 10.1111/jep.70446

**Published:** 2026-04-21

**Authors:** Sophie Macklin, Leanna Woods, Clair Sullivan

**Affiliations:** ^1^ School of Biomedical Sciences, The University of Queensland Brisbane QLD Australia; ^2^ Queensland Digital Health Centre, The University of Queensland Brisbane QLD Australia; ^3^ Metro North Hospital and Health Service Brisbane QLD Australia

**Keywords:** consumer health informatics, digital health, health information exchange, health personnel, rural health services

## Abstract

**Rationale:**

Connected healthcare delivery ensures that the right healthcare information is exchanged with the right healthcare workers, at the right time. Due to the vast distances and dispersed workforce, achieving connected healthcare across rural and remote settings remains a global challenge. Workforce perspectives may help understand how digital transformation can contribute to consumer‐centered healthcare information flow.

**Aims and Objectives:**

To answer the research question: how consumer‐centered is the healthcare information flow across rural and remote health services?

**Method:**

A qualitative study was conducted involving semi‐structured interviews with staff (*n* = 57) from rural and remote healthcare systems (*n* = 6). Transcripts were analyzed to elicit the current state of information exchange between and within health services and associated themes. The findings were evaluated with a subject matter expert and member checking.

**Results:**

The mode of healthcare information flow was largely paper‐based, however frequent use of hybrid (paper and digital records) and digital Clinical Information Systems (CISs) was evident. Transferability of healthcare information could be improved as some systems lack sufficient communication with CISs in other health service types. Resourcing and workforce capabilities were identified to limit transparency of healthcare information. Provided adequate support was available, the rural and remote healthcare workforce support digital transformation and encourage the expansion of digital CISs.

**Conclusion:**

Digital transformation and better integration of CISs can enable a healthcare information journey that is consumer‐centered, ensuring secure, reliable, and efficient exchange of information. Specific recommendations include the expansion of digital health information exchange applications, enhancing local digital infrastructure support, and building a digitally skilled workforce.

## 1 Introduction

Rural and remote populations experience larger health disparities and poorer health outcomes than people in metropolitan areas [1]. Geographic, socioeconomic and workforce factors can amplify barriers to effective healthcare delivery [2, 3]. The exchange of consumer data and health information is critical to ensure healthcare delivery is connected, efficient and secure across care settings, with the potential to improve healthcare, health outcomes and health equity [4, 5].

Evaluating and transforming rural and remote healthcare through digital health has been a strategic focus globally [6, 7]. Exchanging information digitally across healthcare services and providers increases consumer satisfaction as information can be accessed in real‐time [4]. A current example of digital information exchange is the use of electronic medical records, including the national Australian initiative, My Health Record (MyHR) [8].

In this study, Clinical Information Systems (CISs) refers to use of paper, digital or a combination (hybrid) of information systems to exchange consumer healthcare information. The application of CISs to facilitate information exchange in rural and remote settings is currently unknown. To understand the current state from the perspective of rural and remote healthcare workers, an evaluation was undertaken with the research question “how consumer‐centered is the healthcare information flow across rural and remote health services?”

## 2 Methods

A multi‐site qualitative case study design was conducted using semi‐structured interviews. This study followed the Standards for Reporting Qualitative Research (SQRQ) checklist [9] (Supporting Information 1). The current study nests within a larger program of research involving an academic‐led statewide digital health capability assessment completed in 2021 [10, 11].

### 2.1 Setting

The Australian state of Queensland has a geographical area of almost 2 million square kilometers and a population of approximately 5 million [12]. Universal healthcare is delivered by Queensland Health (QH) across acute inpatient care; emergency care; mental health and alcohol and other drug services; outpatient care; prevention, primary and community care; ambulance services, and sub and non‐acute care [13]. Queensland also has cross‐sector partnerships with multiple private hospitals, non‐government organizations, Aboriginal and Torres Strait Islander Community Controlled Health Organizations, general practices, primary health networks, and charitable organizations [14]. There are 16 geographically‐defined healthcare systems across the state that are organized as individual entities, providing decentralized healthcare delivery and accommodation of localized needs [15]. Six of these healthcare systems are classified as rural and remote. Based on geographical remoteness and population size, rural and remote healthcare systems represent a combination of small rural towns, remote communities, and very remote communities as defined by the Modified Monash Model [16]. Two‐thirds of the total First Nations population in Australia and over 38% of Queensland's total population are serviced by rural and remote healthcare systems [17]. Only publicly funded healthcare services were assessed in this study with a focus on healthcare information exchange.

### 2.2 Data Collection

Purposive sampling was employed to select healthcare staff (*n* = 57) from rural and remote healthcare systems (*n* = 6) [11, 18]. Semi‐structured interviews were conducted with an interview protocol tailored to each professional group. The interview guide used in this study was previously developed and tested by HIMSS in a digital health capability assessment [11]. A state‐wide health executive forum enabled the identification of potential participants, with invitations extended to individuals with an understanding of healthcare information exchange across health service types (i.e., hospital, allied health, primary care). Participation was voluntary and accounted for the diverse workforce roles in healthcare services (i.e., clinicians, directors, executives, patient engagement leaders). Reasons for non‐participation was not recorded. Individual interviews were conducted with two interviewers: a Healthcare Information and Management Systems Society (HIMSS) analyst with extensive experience in healthcare consulting and clinical projects (male) and a post‐PhD researcher (female). An additional individual interview with a subject matter expert (SME) was conducted with the same post‐PhD researcher and a research student (female). The interview guide with the SME contained questions aimed at reviewing and validating findings (Supporting Information 2). The SME was purposefully selected due to their extensive knowledge of healthcare information in rural and remote sites, and across different roles (i.e., clinician and executive). Interviewers either had an existing professional relationship with participants based on previous research or developed a professional relationship during study commencement. Participants attained background knowledge of interviewers when consenting to the study. The interviews were conducted and audio‐recorded via web‐based videoconferencing to ensure that nonverbal body language could be considered. Duration of interviews was 30 to 60 min. Interviews were conducted once per participant, unless the participant was involved in more than one healthcare system. Interviews were deidentified using a unique identifier code, manually transcribed verbatim by a transcription service, and verified by a researcher (LW). Prior to analysis, participants were given the opportunity to review their transcripts. Data collection was finalized when no further insights were collected from the diverse range of workforce roles. Member checking [19] with two internal QH staff members was conducted to review accuracy, language and context. Researcher positionality and reflexivity is included in Supporting Information 3.

### 2.3 Data Analysis

A hybrid deductive and inductive thematic analysis approach was conducted to elicit the rural and remote workforce perspectives of healthcare information flow.

### 2.4 Deductive Analysis

Deductive analysis followed the Framework Method [20] and used the text mining software, NVivo (version 12, QSR International) to assist in the coding of the interview transcripts. This involved:

1. Using pre‐defined codes to code the first few transcripts;
2. Developing a working analytical framework on the identified codes;
3. Applying the working analytical framework to the remaining transcripts;
4. Charting the data into the framework matrix and narrative analysis;
5. Narrative analysis of the charted data.

Data was coded and subsequently exported into Microsoft Excel for analysis.

Pre‐defined codes included CISs currently used in rural and remote settings, mode of exchange of healthcare information, sentiment relating to information exchange per health service type, and additional insights relevant to the use of CISs and healthcare information flow in rural and remote were coded to a separate parent code.

Sentiment analysis identifies peoples' opinions, attitudes and emotions towards a specific subject [21]. Coded data was defined as null (data of no sentimental value), positive, negative, or mixed sentiments according to the perception of different CISs used to facilitate the flow of healthcare information [22].

### 2.5 Inductive Analysis

Working with text segments within the ‘additional insights’ code, inductive analysis was conducted to further clarify the enablers, barriers and provide contextual understanding of healthcare information exchange in rural and remote settings. This process also ensured that relevant coding segments not captured in the deductive analysis phase were incorporated into the final findings.

The SME interview transcript underwent qualitative analysis using the same process of analysis in NVivo. Additional information was added to the relevant data tables and integrated into the findings. No adjustments were made to the analysis beyond clarification of acronyms, software and context‐specific terminology after member checking by QH.

All coding was completed by SM and verified by a second senior researcher (LW). Data was stored on a secure data management system with access provided only to approved investigators.

### 2.6 Ethics Approval

The research was conducted according to the National Statement on Ethical Conduct in Human Research [23], received multisite ethics approval from the Royal Brisbane and Women's Hospital [HREC/2020/QRBW/66895] and site‐specific research governance approvals from all sites. Consent was provided prior to data collection.

## 3 Results

### 3.1 Modes of Healthcare Information Exchange

A review of CISs currently in use at rural and remote sites identified similarities and disparities of system usage across and between the health services (Table 1). Additional speciality systems may be used in rural and remote healthcare settings, however the main CISs are included. Sentiment of healthcare exchange across health services is described (Table 2).

**Table 1:** Summary of clinical information systems (CISs) used in different health service types in rural and remote sites.

	Clinical information system	
Type of Health Service	Public healthcare	Private healthcare
Allied health	Paper‐based CIMHA	Hybrid
Diagnostics	Paper‐based Auslab Pathology Auscare (Pathology) PACS QRiS	Paper‐based with limited CPOE
General practice Primary care	Best Practice Medical Director Communicare HBCIS EDIS	Best Practice
Hospital Emergency department Outpatient department	Paper‐based HBCIS EDIS The Viewer Communicare	N/A
Indigenous health	Hybrid paper and digital Medical Director Best Practice Medical Director Communicare HBCIS EDIS	N/A
Medical specialist	Paper‐based The Viewer Communicare	Medical Director
Pharmacy	Paper‐based iPharmacy	Digital prescribing from general practitioners
Queensland ambulance service	Hybrid paper and digital Electronic Ambulance Report Form (eARF)	N/A
Residential aged care	Leecare	Hybrid
Retrieval services Queensland	Medical Director (own version) EMR (own version)	N/A

*Note:* N/A indicates no data representing the clinical information system in that health service type.

Abbreviations: CIMHA, consumer integrated mental health and addiction; CPOE, computerized provider order entry; EDIS, emergency department information system; EMR, electronic medical record; HBCIS, hospital‐based corporate information system; PACS, picture archiving and communication system; QRiS, queensland radiology information system.

**Table 2:** Sentiment analysis of clinical information systems (CISs) used in different health service types in rural and remote sites.

	Clinical information systems		
Health service type	Paper	Hybrid	Digital
Allied health	N/A	—	+
Diagnostics	N/A	—	+
General practice	—	—	+/−
Hospital	—	+/−	+/−
Emergency department			
Outpatient department			
Indigenous health	N/A	—	—
Medical specialist	N/A	0	N/A
Mental health	N/A	N/A	+/−
Pharmacy	N/A	N/A	+/−
Queensland ambulance service	—	—	0
Residential aged care	N/A	N/A	—
Retrieval services Queensland	—	—	—

KEY: 0, null sentiment; +, positive sentiment; −, negative sentiment; +/−, mixed positive and negative sentiment. N/A indicates no data available for that health service type.

### 3.2 Paper‐Based Healthcare Information Exchange

As rural and remote health services initiate digital transformation, paper‐based methods are at the foundation of healthcare information exchange. Participants expressed negative sentiment due to increased information duplication, and difficulty of real‐time and correct information transfer to other health services. Accessing patient data can also require multiple processes and electronic systems across and within the different health services in rural and remote sites.

For allied health professionals who service multiple health services, it is common to capture healthcare information from a variety of sources. Participants identified that visiting allied health staff would also use their own systems as, “the only solution we could come up with that was joined up was everyone prints stuff out and puts it in a paper chart.” (B009).

Diagnostic services and pharmacy are categorized as paper‐based, with participants emphasizing that pathology completed outside the public system is still transferred through paper‐based processes. Participants detailed the physical process of sending charts to ensure information is exchanged between the health services, “…if there's a corresponding chart in [city] and we'll ask them to… physically send the chart …” (F002).

General practitioners (GPs) transferred healthcare information via methods such as fax and regular post, causing considerable issues for the health service, “… the patient is calling in saying “hey, my GP hasn't got this, I'm here sitting in an appointment, it's urgent, can you please send us…” and they say, “oh we've sent it” … we get so many complaints” (D008).

### 3.3 Hybrid Healthcare Information Exchange

Hybrid mode of information exchange has been adopted in rural and remote settings. Participants indicated that utilization of a hybrid exchange requires access to more than one CIS to collect information. The workforce is adversely affected when using multiple information systems due to untimely uploading and access to patient information, particularly discharge summaries.

In allied health settings, participants use both paper‐based and digital systems. Patient notes are often documented on paper and subsequently entered into a digital CIS to allow transparency of consumer information in the hospital setting. “…we will handwrite most of our notes …. then write it on the [CIS] so that the GPs who are attached to the [town] Hospital … can see from day‐to‐day… “ (B005). Ambulance services utilize different modes of information exchange dependent on the service. One site described the process of collecting and transferring patients and their information via a community clinic and Queensland Ambulance Service (QAS), both which use different CISs:

When I get there, I can photocopy [my notes], hand over that piece of paper [to QAS] …. If, however, I ask the QAS, halfway meet,… [I] have two copies so that on the way I can document any information, … I will hand that to the ambulance … they then do it on a tablet, then they can print off at the hospital and handover their information.

(B005)

In hybrid and digital modes of information exchange, participants have indicated that there is some level of information exchange that is advantageous. The workforce in rural and remote sites may work across GP and hospital settings, providing access to clinical records. “We're very lucky here because all of the doctors work in both the hospital and the GP practice, so we can access GP notes from the hospital and hospital notes from the GP practice, …” (C007).

### 3.4 Digital Healthcare Information Exchange

Clinicians are adopting digital CISs so that more healthcare professionals can track the consumer's journey. Information can be more easily collected for consumers receiving treatment in a health service utilizing digital CISs as it can facilitate efficient upload and access to information exchange platforms.

Primary care practitioners utilize these systems to gather data on patients including discharge summaries and other diagnostic results. However, transferring information from the GP using a digital CIS to another health service is a barrier for clinicians. “… you may go to the general practice in [town], which is ours, but then you'll present to the hospital, which is ours, and we can't get that record because [CIS] sits on the practice…” (C005).

Participants are satisfied with the practice of using digital CISs in diagnostics and allied health. Particularly for diagnostic services, clinicians have detailed the positive impacts of this digital exchange, “when we went to a digital application in radiography… [it] undoubtedly improved patient outcomes in early diagnosis.” (C001).

### 3.5 Additional Insights

Further insights on the current and ideal future states of healthcare information exchange in rural and remote areas emerged from participants. Three major themes were identified. These include: (1) interoperability and reliance on paper‐based systems, (2) access to infrastructure and resources and, (3) healthcare staff capabilities.

#### 3.5.1 Interoperability and Reliance on Paper‐Based Systems

The most prominent insight was the use of multiple systems required between different health services to piece together the consumer journey. Lack of interoperability was largely associated to the production of data silos at each location, particularly with external health providers and NGOs. Patient safety was a concern when CISs were unable to communicate, “We have so many electronic programs that don't talk to each other that create barriers and sometimes gaps, sometimes patient safety risk….” (D009).

Participants expressed hearing consumers' frustration at the repeated requests to provide the same information, “…they get sick of saying the same thing, again and again and again…” (F011) To enable better interoperability in rural and remote sites, participants have proposed a “one patient, one record”, ideally obtaining a complete history of the consumer to enable better quality of care.

While paper‐based systems are at the foundation of information transfer, converting into hybrid and digital models of exchange have been identified to create additional barriers for clinicians:

[CIS 1] was put in [some sites] by doctors. … [CIS 2] has been put in [other sites]… where nurses, health workers, allied health can use it, but the doctors hate it. So, half the doctors didn't have one, so they love it because they're on paper.

(E002)

Overall, participants are eager to digitally transform their healthcare services, stating major barriers to using a paper‐based system including tracking down and losing charts, extensive filing and administrative work, and decreased workflow efficiency.

The label of “interoperability” was not considered accurate when describing the sharing of healthcare information. “Information visibility and exchange…” (G001) was offered as the more relatable phrase. To enable better visibility, clinicians desire information to flow through digital CISs seamlessly by feeding into a singular CIS.

Future aspirations for healthcare information exchange include the use of electronic prescribing and increased feeding of information into relevant exchange platforms to ensure accessibility and visibility across all health service types. This includes access to electronic report forms and any information documented by the RFDS, whether that entails exchange of summaries or integration into current systems.

#### 3.5.2 Access to Infrastructure and Resources

Digital transformation requires access to resources including funding, adequate ICT infrastructure and staff training, “We've got sites that are still running on 1.5MB copper cabling.” (B001) If these barriers are accurately addressed, the future state includes clinicians having, “… infrastructure within our hospital to be able to deal with that much internet connection and the busyness of the connection.” (D004). Inadequate ICT infrastructure and training also hindered the collection and safe keeping of patient records, “When the local [health service] had a hacking with a ransomware attack they lost basically a month's worth of records… they didn't have properly secured and back up servers…”. (C006).

The key theme surrounding access to infrastructure and resources in rural and remote settings was, “… Connectivity, connectivity, connectivity…” (G001). The participant described a township which had, “… 2 weeks without mobile connectivity…” (G001) due to barriers in communication technology such as satellite and mobile connectivity and extreme weather conditions. Limited bandwidth of health services was mentioned at some healthcare sites, “… can't run more than one telehealth consultation at one time” (G001). Receiving funding to invest and deliver ICT infrastructure in a timely manner to health services was critical.

#### 3.5.3 Healthcare Staff Capabilities

Some participants expressed that they are more comfortable with paper‐based systems than digital, as one detailed that, “We've got digital refugees,… [they've] come to the bush, because you don't have [an EMR]” (B001). Low digital literacy and an aging workforce have also increased the negative perception of digital transformation, however when provided with sufficient support, clinicians indicate that these are no longer barriers.

Capabilities of healthcare staff in terms of digital exchange of healthcare information varies considerably. The training involved with increasing the skillset of healthcare workers is perceived as burdensome in these regions. The requirement for and retention of non‐clinical management staff to support digital health is difficult as, “the minute I grow my own [staff] and they become good performers, they'll be gone to [metropolitan city]” (G001).

Similarly for the uptake of MyHR, support is needed for healthcare providers to integrate the system into everyday practice, “… the training that is required… everyone has a desire to participate,… but we just aren't resourced” (G001). The result of this is primary healthcare centers sometime avoiding MyHR uploads, “… although they have the capability.” (G001) Digital training of healthcare providers is critical to prevent unnecessary risks and ensure security and governance of healthcare information.

## 4 Discussion

There is a need to shift healthcare information exchange from health service‐centered to consumer‐centered in rural and remote settings. The introduction of digital CISs has presented issues that are not unique only to these regions, with information duplication and generation of data silos evidenced globally [24]. Current barriers to digital transformation continue to limit information exchange. Most significant challenges include connectivity issues and lack of ICT infrastructure, and appropriate education and training of the workforce, specifically in digital literacy, data security and governance. Collectively, these findings indicate the areas for improvement for healthcare information flow in rural and remote settings and include an idealized future state.

Study findings emphasized the need for easily accessible and comprehensible health information exchange to healthcare providers across all health service types to ensure information is available when and where it is needed [25, 26]. Progressing digital transformation in rural and remote settings to reduce the prevalence of paper information exchange has been evidenced to reduce duplication, errors and misinformation and match the flow of information with the consumers' journey [4, 27, 28]. As digital CISs use increases, real‐time uploading of healthcare information as consumers move across health services can be facilitated, promoting continuity of care and strengthening health outcomes [4, 29]. Connectivity issues and lack of ICT infrastructure support currently impede this exchange [30], with limited and outdated capabilities reducing access and efficiency of service [31]. Enhancing connectivity will enable safer and real‐time access to information, and implementing dedicated local ICT support will ensure timely repair, replacement and troubleshooting of technology and connectivity issues [28], promoting consumer‐centered information exchange [32]. To ensure uptake and sustainability of this digital transformation, appropriate education and training of the healthcare workforce will be required to enhance digital literacy, data security [4, 33, 34], especially as these settings deliver healthcare through different organizations.

The future state of healthcare information exchange has been idealized as using a centralized, consumer‐centered digital application, placing value on improving healthcare outcomes [35], consumer and provider experience. Reduction of paper‐based healthcare delivery and uptake of electronic records will enable improvement in clinical workflow processes by providing real‐time patient data, regardless of time or location [4]. As digital transformation progresses throughout rural and remote settings, ensuring accessibility to continued education and professional development programs in rural and remote settings will be important for workforce retention, increasing digital capability and ensuring health equity and quality of care [36].

To address the current barriers and challenges, three key recommendations are proposed to enhance healthcare information exchange across rural and remote healthcare settings. A suggested approach to each recommendation is presented to improve consumer‐centered information exchange (Table 3). These recommendations can be used to provide guidance to policymakers and healthcare executives when undertaking digital transformation across rural and remote settings.

**Table 3:** Recommendations from this research for enhancing healthcare information exchange in rural and remote sites.

Recommendation	Approach
1. *Expand use of digital health information exchange applications* Improving accessibility for consumer information across healthcare settings, particularly for less digitally enabled sites	Increase awareness and support for adoption of CISs
2. *ICT infrastructure support* Increasing local ICT support to facilitate consumer information exchange and progress digital transformation	Infrastructure upgrades are already underway
3. *Develop a digitally skilled workforce* Improving digital literacy to enable increased acceptance and use of digital CISs and information exchange across healthcare settings	Increase digital capabilities of healthcare providers through investment in education, training and support

### 4.1 Limitations

Consumer interviews were not conducted in this study. The transparency of consumer health information transferred to, from, and within the private health sector was not specifically examined using the study methods, with all research participants public healthcare employees. These limitations may be addressed through future research.

## 5 Conclusion

There is a need for consumer‐centered healthcare information exchange in rural and remote settings. Three key recommendations have been proposed to improve information exchange including: increasing uptake of digital health applications and ICT infrastructure support and facilitating workforce upskilling in digital health. Enabling the flow of healthcare information to match the journey of the consumer will be essential to improving outcomes for consumers, clinicians, and the population at large. Identifying how healthcare information is currently exchanged, this study provides policy‐level guidance to advance and achieve consumer‐centered information exchange and strengthen healthcare delivery across rural and remote settings.

## Conflicts of Interest

The authors declare no conflicts of interest.

## Supporting information

Supporting File 1

Supporting File 2

Supporting File 3

## Data Availability

The data that support the findings of this study are available on request from the corresponding author. The data are not publicly available due to privacy or ethical restrictions.
