# Supplementary material for: The Flow of Healthcare Information in Rural and Remote Settings: A Qualitative Approach
Source: J Eval Clin Pract. 2026 Apr 21;32(3):e70446. doi: 10.1111/jep.70446 (PMC13099109; doi:10.1111/jep.70446)
Supplement: Supplementary file 2 — Supporting File 2 [file JEP-32-0-s002.docx]

# **Supporting Information 2 – Subject matter expert (SME) interview guide**

1. Talk me through the healthcare journey of consumers. How does this compare to the journey of their health information?

2. Do you have any insight into the consumer experience on the topic?

3. Can you walk me through how health care information is currently stored in health services across your site?

4. What are some potential barriers to the flow of health information?

5. What about enablers to the flow of consumer health information?

6. What other external health service providers do you use in rural and remote Queensland/your site?

7. How can the journey of consumer health information be improved?

8. Are there any initiatives in place to improve patient health information flow?

9. Do you have an understanding of the consumer information flow across pharmacy/specialist/aged care/QAS/mental health?

10. Can you describe the information flow between Queensland Health facilities and private facilities?
