# Supplementary material for: The Flow of Healthcare Information in Rural and Remote Settings: A Qualitative Approach
Source: J Eval Clin Pract. 2026 Apr 21;32(3):e70446. doi: 10.1111/jep.70446 (PMC13099109; doi:10.1111/jep.70446)
Supplement: Supplementary file 3 — Supporting File 3 [file JEP-32-0-s001.docx]

# **Supporting Information 3 – Researcher Positionality and Reflexivity**

The research team consisted of a post-PhD digital health researcher, a clinician-academic specialising in digital health and digital transformation, and a research student. The collective background of the team may have influenced the conceptual framing of the study and their perspectives on rural and remote digital capability. Reflexivity and academic rigor was maintained through discussion of data interpretation with subject matter experts outside the research team, defining the coding framework and subsequent themes collaboratively, and verification of coding by a second researcher.
